# Complete genome sequences of *Klebsiella pneumoniae*, *Klebsiella quasipneumoniae*, and *Klebsiella variicola* clinical isolates from an epidemiology study

**DOI:** 10.1128/mra.01060-24

**Published:** 2025-02-12

**Authors:** Pedro Peralta-Macotela, Bibiana Flores-Monzón, Karla Isabel Lira de León, Ana Alicia Sánchez-Tusie, Nadia Rodríguez-Medina, Alejandro Alvarado-Delgado, Edgar Aguilar-Vera, Federico Zumaya-Estrada, Ulises Garza-Ramos, Maria Carlota García-Gutiérrez

**Affiliations:** 1 Laboratorio de Epidemiología Traslacional, Centro de Investigación Biomédica Avanzada, Facultad de Medicina, Universidad Autónoma de Querétaro, Santiago de Querétaro, Querétaro, México; 2 Facultad de Química, Universidad Autónoma de Querétaro (UAQ), Querétaro, Querétaro, México; 3 Centro de Investigación Sobre Enfermedades Infecciosas (CISEI), Grupo de Investigación y Docencia en Resistencia Bacteriana, Instituto Nacional de Salud Pública (INSP), Cuernavaca, Morelos, México; Loyola University Chicago, Chicago, Illinois, USA

**Keywords:** *Klebsiella pneumoniae *species complex, KpSC, healthcare-associated infections, opportunistic bacterial pathogens

## Abstract

An epidemiology study of *Klebsiella spp*. causing infections was carried out. In the study, *K. pneumoniae* was identified with a prevalence of 94.6%, *K. quasipneumoniae* with 3.8%, and *K. variicola* with 1.6%. Here, we report the draft genome sequence of four selected *Klebsiella pneumoniae* species complex (KpSC) clinical isolates obtained from different sources.

## ANNOUNCEMENT

The main *Klebsiella pneumoniae* species complex (KpSC) phylogroups include *K. pneumoniae*, *K. quasipneumoniae*, and *K. variicola* (1). These bacteria are the most frequently reported as causing hospital- and community-associated infections (2). They are the most frequently associated with urinary tract infections (UTIs), pneumonia, septicemia, and wound and soft tissue infections in healthcare settings (3). Also, the gastrointestinal carriage of *K. pneumoniae* was observed as a reservoir for healthcare-associated infections (4). Here, we present the genomes of four bacterial species from KpSC obtained from different origins.

The faculty of the Medicine of Autonomous University of Queretaro carried out an epidemiology study of *K. pneumoniae* infections in three public hospitals at Queretaro State from November 2016 to December 2019 (Authorization SESEQ-1053). We collected 130 *K. pneumoniae* clinical isolates identified by Vitek 2 manufacturer. The bacterial species were determined using PCR multiplex (5). *K. pneumoniae* was identified with a prevalence of 94.6%, followed by *K. quasipneumoniae* with 3.8%, and *K. variicola* with 1.6%. In this study, we performed whole genome sequencing of the four *Klebsiella* sp. isolates (Table 1). A single colony of isolates was grown on LB agar and then inoculated in LB broth at 37°C with shaking at 200 rpm overnight. Genomic DNA was extracted with the MasterPure complete DNA (MC85200; Epicentre). Illumina MiSeq libraries were constructed (Illumina, Inc., San Diego, CA, USA) using the Nextera XT Sample Prep Kit (Illumina, Inc.) and sequenced on standard flow cells (2 × 150). Illumina reads were quality verified by FastQC software v0.11.8 (https://www.bioinformatics.babraham.ac.uk/projects/fastqc/) and trimmed with Trim_Galore v0.6.4 (https://www.bioinformatics.babraham.ac.uk/projects/trim_galore/) using the following parameters: --illumina, --paired, --phred33, --fastqc, --retain_unpaired, and --length 20. The *de novo* assembly of *Klebsiella* spp. genomes with Illumina reads was performed using SPAdes Genome Assembler (v3.6.0) software using the parameters for paired-end libraries and the following pipeline options: --careful, --only-error-correction, and --continue. All genomes were annotated using the National Center for Biotechnology Information (NCBI) Prokaryotic Genome Annotation Pipeline (PGAP) (v4.2.0) (https://www.ncbi.nlm.nih.gov/genome/annotation_prok/) (6).

**TABLE 1:** Clinical, epidemiological, and genome data of *Klebsiella pneumoniae* species complex

	Bacterial specie			
	*K. pneumoniae*		*K. quasipneumoniae subsp. similipneumoniae*	*K. variicola subsp. variicola*
Strains	Kpn_211	Kpn_246	Kq_88	Kv_80
Origin	bronchial secretion	wound discharge	rectal swab	rectal swab
Year of isolation	2019	2019	2017	2017
Age	57	36	58	49
Sex	female	female	male	male
Symptomatic or asymptomatic	Symptomatic	Symptomatic	Symptomatic	Symptomatic
Service	Internal medicine	Surgery	Traumatology	Internal Medicine
Genomic characteristics				
Protein coding	5260	5263	5203	5341
tRNA	81	81	80	84
rRNA	29	23	29	32
C + G content (%)	57.25	57.28	57.75	57.0
Total raw reads	21,766,936	21,710,744	22,518,502	22,102,626
Total size (bp)	5537657	5537638	5488623	5705612
N50	449876	780210	326018	425961
Number of contigs	84	76	157	55
GenBank accession no.	JBGRWH000000000	JBGRWG000000000	JBGRWF000000000	JBGRWE000000000
SRA database	SRR31547390	SRR31547389	SRR31547388	SRR31547387
Genetic characteristics				
Capsular serotype	unknown	KL15	KL53	KL55
Resistome	*rmtC*, *aph(6)-Id*, *aph(3'')-Ib*, *aac(3)-Iia*, *aac(6')-Ib-cr*, *fosA*, *catB3*, *oqxB*, *oqxA*, *qnrB1*, *sul1*, *sul2*, SHV-11, NDM-1, TEM-1B, CTX-M-15, OXA-1	*aph(6)-Id*, *aph(3'')-Ib*, *aac(3)-IIa*, *aac(6')-Ib-cr*, *fosA7*, *fosA6*, *catB3*, *oqxB*, *oqxA*, *qnrB1*, *sul2*, *tet(A)*, *dfrA14*, SHV-89, CTX-M-15, TEM-1B, OXA-1	*fos*A, *oqxB*, *oqxA*, OKP-B-19	*fos*A, *oqxB*, *oqxA*, LEN-13
Virulome	*mrKABCDFHI*, *entABC*, *ureABDFG*	*mrKABCDFHIJ*, *entABC*, *ureABDFG*, *kfuABC*	*mrKABCDFHIJ*, *entABC*, *ureABDFG*, *kfuABC*	*mrKABCDFHIJ*, *entABC*, *ureABDFG*, *kfuABC*
Incompatibility groups	IncFIB(K), IncFII(K), IncFII(Yp), Col440I and ColpVC	IncFIA(HI1), IncFIB(K), and IncFII(K)	IncFIB(K)	IncFIB(K), IncFII(K) and Col440I

Table 1 contains the information of strains and genomic and genetic characteristics. The full genome was analyzed for predicted antibiotic resistance genes and incompatibility groups using ResFinder 4.1 (7). A clear difference in the resistome was observed among KpSC genomes from the study. The β-lactamases corresponding to their chromosomes were identified as SHV-11, OKP-B-19, and LEN-13 alleles, respectively. The predicted virulence-associated genes were shared between the different genomes; however, the *kfuABC* operon was identified only in Kpn_246, Kq_88 and Kv_80 isolates (Table 1).

For *K. pneumoniae* (8) and *K. variicola* (9), a multilocus sequence typing of total-genome-sequenced bacteria was used. The ST273 and ST1876 were identified for *K. pneumoniae* Kpn_211 and Kpn_246 genomes, and ST317 for *K. variicola* Kv_80 genome. Using Kleborate v2.3.2 (10), the capsular serotype was identified (Table 1). The plasmids of the incompatibility groups were identified using PlasmidFinder (11); the IncFIB(K) and IncFII(K) were shared among KpSC genomes from the study (Table 1).

## Data Availability

This Whole Genome Shotgun project has been deposited in DDBJ/ENA/GenBank under accession no. PRJNA1148126. See also Table 1.
